# Retraction: Exploring the role of gut microbiota in autoimmune thyroid disorders: a systematic review and meta-analysis

**DOI:** 10.3389/fendo.2026.2023216

**Published:** 2026-10-15

**Authors:** Frontiers Editorial Office

The journal retracts the article cited above.

Following concerns regarding the originality of the article, an investigation was conducted in accordance with Frontiers’ policies. It was found that the complaint was valid and that the article should be retracted, because of an unacceptable level of similarity to another article: Sawicka-Gutaj, N.; Gruszczyński, D.; Zawalna, N.; Nijakowski, K.; Muller, I.; Karpiński, T.; Salvi, M.; Ruchała, M. Microbiota Alterations in Patients with Autoimmune Thyroid Diseases: A Systematic Review. Int. J. Mol. Sci. 2022, 23, 13450. https://doi.org/10.3390/ijms232113450.

This retraction was approved by the Chief Executive Editor of Frontiers. The authors received a communication regarding the retraction and had a chance to respond. This communication is recorded by the publisher.

Frontiers would like to thank Dawid Gruszczyński for contacting the journal regarding the published article.

